# Transendothelial Migration Enables Subsequent Transmigration of Neutrophils through Underlying Pericytes

**DOI:** 10.1371/journal.pone.0060025

**Published:** 2013-03-26

**Authors:** Chantal E. Ayres-Sander, Holly Lauridsen, Cheryl L. Maier, Parid Sava, Jordan S. Pober, Anjelica L. Gonzalez

**Affiliations:** 1 Department of Biomedical Engineering, Yale University, New Haven, Connecticut, United States of America; 2 Department of Immunobiology, Yale School of Medicine, New Haven, Connecticut, United States of America; UAE University, Faculty of Medicine & Health Sciences, United Arab Emirates

## Abstract

During acute inflammation, neutrophil recruitment into extravascular tissue requires neutrophil tethering and rolling on cytokine-activated endothelial cells (ECs), tight adhesion, crawling towards EC junctions and transendothelial migration (TEM). Following TEM, neutrophils must still traverse the subendothelial basement membrane and network of pericytes (PCs). Until recently, the contribution of the PC layer to neutrophil recruitment was largely ignored. Here we analyze human neutrophil interactions with interleukin (IL)-1β-activated human EC monolayers, PC monolayers and EC/PC bilayers *in vitro.* Compared to EC, PC support much lower levels of neutrophil binding (54.6% vs. 7.1%, respectively) and transmigration (63.7 vs. 8.8%, respectively) despite comparable levels of IL-8 (CXCL8) synthesis and display. Remarkably, EC/PC bilayers support intermediate levels of transmigration (37.7%). Neutrophil adhesion to both cell types is Mac-1-dependent and while ICAM-1 transduction of PCs increases neutrophil adhesion to (41.4%), it does not increase transmigration through PC monolayers. TEM, which increases neutrophil Mac-1 surface expression, concomitantly increases the ability of neutrophils to traverse PCs (19.2%). These data indicate that contributions from both PCs and ECs must be considered in evaluation of microvasculature function in acute inflammation.

## Introduction

Neutrophil recruitment from the blood to the tissue is a central component of the acute inflammatory response. This process begins with changes in the endothelial cell (EC) lining of post-capillary venules, notably increased expression of leukocyte adhesion molecules (LAMs) and increased display of neutrophil-activating chemokines. LAMs such as P- and E-selectin mediate the initial tethering and subsequent blood flow-dependent rolling of neutrophils on the endothelium. Rolling leukocytes encounter and respond to the surface-bound chemokines, principally interleukin-8 (IL-8 or CXCL8) in humans, triggering firm attachment via integrin-mediated adhesion to other endothelial LAMs, most significantly to intercellular adhesion molecule-1 (ICAM-1 or CD54) by neutrophil Mac-1 (CD11b/CD18) [1], [2]. EC chemokines and ICAM-1 then contribute to neutrophil crawling along the EC surface and eventually to initiation of transendothelial migration (TEM) that occurs at or near inter-EC junctions. Other EC adhesion molecules at the junctions, such as platelet-endothelial cell adhesion molecule (PECAM-1 or CD31) and CD99, participate in transendothelial migration but not in the initial neutrophil adhesion or activation [3]. Once the EC monolayer has been breached, the neutrophil still must cross the subendothelial basement membrane and a network of pericytes (PCs) that reside within the basement membrane. While the details of capture and traversing the EC lining of the venule have been extensively studied for several decades using *in vitro* models, as well as *in situ* observations, much less attention has been paid to the final events involving neutrophil interactions with PCs [1], [4]–[7].

PCs are large, elongated cells that provide structural integrity to capillaries and post-capillary venules. They contribute to the formation of the subendothelial basement membrane and interact with ECs in a number of ways that are important for the maturation and maintenance of the microvascular system [6], [8]–[10]. In particular, the PC serve to inhibit EC proliferation, collaborating with ECs to generate active transforming growth factor beta (TGF-β) and reducing EC permeability through production of angiopoietin-1 (Ang-1) [10]–[12]. The density of PCs in the microvasculature is both tissue- and vessel-dependent with EC:PC ratios ranging from 1∶1 to 10∶1; PCs are most numerous in the venous capillaries and post-capillary venules of the eye, lung, heart and skin [8], [9], [13], [14]. It has recently been shown that PCs actively influence neutrophil extravasation *in vivo* although little is known about the molecular details of this process [6], [7]. Further analyses of the interactions of this cell type with neutrophils using *in vitro* models will likely lead to better understanding of and perhaps to identification of new therapeutic targets for modifying acute inflammation.

The analysis of EC interactions with neutrophils has profited greatly from the culture of human EC described over 35 years ago [15]. We recently described a method for isolating and culturing human PCs from placental microvessels [12], providing a readily renewable source of this cell type for functional studies comparable to the availability of human ECs. To address the relative lack of information about the function of PCs in acute inflammation, we have analyzed and compared the interactions of freshly isolated human peripheral blood neutrophils with human umbilical vein EC monolayers, human placental microvascular PC monolayers and EC/PC bilayers. Specifically, we have examined how interleukin-1β (IL-1β)-activated EC or PC cultures influence neutrophil functional activities (adhesion, motility, polarization, transmigration) using time-lapse video microscopy. We have specifically analyzed the roles of ICAM-1 and IL-8 expression by ECs and PCs, the principal adhesion molecule and chemokine, respectively, which regulate human neutrophil recruitment. As previously observed IL-1β-activated ECs function to facilitate neutrophil transmigration [16]–[19], whereas similarly activated PCs, either as a monolayer or as part of an EC/PC bilayer, function to limit neutrophil transmigration. This difference appears to largely result from limited expression and ineffective localization of ICAM-1 for neutrophil binding to PCs. Transduction of PCs with ICAM-1 can enhance neutrophil binding but is not on its own sufficient to promote transmigration. Significantly, the process of transendothelial migration primes neutrophils to transmigrate through a PC layer, in part by increasing the expression of Mac-1 and other CD18 integrins that serve as potential ligands for ICAM-1 and basement membrane proteins. Our findings suggest that EC/PC bilayers can be used to more accurately model the process of neutrophil diapedesis.

## Materials and Methods

### Ethics Statement

The use and attainment of human cells were approved by Yale University Human Investigation Committee (HIC) of the Internal Review Board (IRB) as part of the Human Research Protection Program. This research was conducted in accordance with approved protocols and in line with the standards set by the Helsinki Declaration. All advertisements for volunteers were approved by the Yale University HIC IRB and written informed consent was obtained from all human volunteers prior to blood collection. Data collection and analyses were performed anonymously.

### EC and PC Culture and Activation

Human vascular cells were isolated from discarded, de-identified umbilical cords and placentas under protocols approved by the Yale Human Investigation Committee. Human umbilical vein ECs were harvested using collagenase as described by Gimbrone *et al.* [20] and serially subcultured in flasks coated with 0.1% gelatin (Sigma-Aldrich, St. Louis, MO) in M199 (Gibco, Grand Island, NY) medium supplemented with 20% FBS (Hyclone Laboratories, Inc., Logan, UT), 1% penicillin-streptomycin (Gibco), 0.1 mg/ml heparin and 50 µg/ml endothelial cell growth supplement (ECGS) (Collaborative Biomedical Products, Bedford, MA) as described by Thornton *et al*. [21] Human placental PCs were isolated by explant outgrowth from human placental microvascular segments obtained by collagenase digestion of placenta according to the methods described in Maier *et al*. [22] and serially subcultured in M199 medium supplemented with 20% FBS and 1% penicillin-streptomycin. Serially passaged ECs prepared in this way uniformly express the EC marker CD31 and are devoid of CD45-expressing leukocytes while the cultured PCs uniformly express Thy-1 and NG2 as well as smooth muscle contractile proteins and lack both CD31 and CD45. To activate vascular cells, ECs or PCs were cultured on gelatin-coated 25 mm circular coverslips (VWR International) until visually confluent followed by addition of 10 U/ml IL-1β (PeproTech, Rocky Hill, NJ) for 4 or 24 hr as indicated. IL-1β dose was chosen based on pilot studies of IL-1β activation efficacy.

### Retroviral Transduction of PCs

PCs were transduced using retroviral supernatant from Phoenix packaging cells transfected with a pLZRS vectors containing an ICAM-1 insert and cytomegalovirus promoter as previously described in detail by Clark et al. [23], [24]. The Phoenix packaging cell line were derived and served as the source of retroviral stocks as previously described by Zhang et al. (provided as a generous gift by Dr. G. P. Nolan, Stanford University, Palo Alto, CA) [25]. Retroviral supernatant was collected from the Phoenix cells, syringe filtered and supplemented with 8 µg/mL polybrene (American Bioanalytical, Natick, MA) to improve retroviral infection. Over a 5 day interval, Phoenix supernatant was added to PC culture flasks daily for 4 hr, then removed and replaced with fresh PC medium. ICAM-1-transduced PCs (ICAM-1+PC) were then positively sorted for elevated ICAM-1 expression using a Becton Dickinson FACS Aria. When used for adhesion, transmigration, and IL-8 detection, ICAM-1 transduced PCs were activated with10 U/ml IL-1β for 4 hours.

### ICAM-1 Expression on Vascular Cells

ECs or PCs were cultured on gelatin-coated 25 mm circular coverslips until confluent and activated with IL-1β for the indicated times. For FACS analysis, cells were removed from coverslips with trypsin+EDTA/HBSS, incubated with primary mouse anti-human ICAM-1 (CD54) antibody (R&D Systems) or a normal mouse isotype-matched IgG control (R&D Systems) followed by an anti-mouse IgG-FITC secondary antibody (Sigma-Aldrich), rinsed in PBS, fixed in a 2% paraformaldehyde/PBS solution and analyzed using a BD LSRII flow cytometer. ICAM-1 expression was analyzed using FlowJo software (Tree Star, Inc). For confocal fluorescence imaging, samples were fixed in 2% paraformaldehyde as a monolayer, stained using the antibodies described above, and examined using a Zeiss LSM 510 confocal microscope.

### Interleukin-8 Expression

Interleukin-8 (IL-8 or CXCL8) secretion was measured using a human IL-8 ELISA Ready-SET!-Go kit (eBioscience, San Diego, CA). EC or PC monolayers or EC/PC bilayers were cultured on 3.0 µm Transwells in 24 well plates until confluent. Mono- or bilayers were then activated with IL-1β for 4 hr. Media was collected and assayed with the IL-8 ELISA kit using the provided protocol. For imaging of bound IL-8, ECs or PCs were cultured on 25 mm circular coverslips until confluent and activated with IL-1β for 4 hr. EC or PC cultures were then fixed in 2% paraformaldehyde and stained with primary anti-human IL-8 antibody (eBioscience) followed by anti-mouse IgG-TRITC secondary antibody (Sigma-Aldrich). Samples were imaged using a Nikon Eclipse TE2000-U microscope and analyzed using ImageJ (NIH).

### Neutrophil Isolation

Neutrophils were obtained from venous blood drawn from healthy human volunteers under a protocol approved by the Yale Human Investigation Committee, with written consent given by each volunteer. Each blood sample was drawn into a syringe containing citrate-phosphate-dextrose (Sigma-Aldrich) and a 6% HMW Dextran (Polyscience) solution. The whole blood was allowed to stand for 45 min, after which the plasma was drawn off and the remaining cell suspension was centrifuged at 1200 RPM. Following centrifugation, the supernatant was removed and the pellet resuspended in PBS and then added to Histopaque 1077 (Sigma-Aldrich). The cell-Histopaque suspension was then centrifuged at 1200 RPM for 20 min. The neutrophil-rich pellet was resuspended in PBS and centrifuged to remove any remaining Histopaque. The resulting pellet was resuspended in PBS containing calcium, magnesium and 6% glucose at a concentration of 1×10^6^ cells/ml, based upon hemacytometer counts.

### Neutrophil Adhesion

To assess neutrophil adhesion, freshly isolated (naive) neutrophils (at a concentration of 1×10^5^ cells/ml) were injected into a Sykes-Moore adhesion chamber containing confluent EC or PC monolayers on coverslips, previously activated or not with IL-1β, as indicated, and allowed to adhere for 500 sec. The number of neutrophils in contact with the monolayer was counted and the chamber was inverted for an additional 500 sec. The chamber was then placed upright and the neutrophils remaining in contact with the monolayer were counted and expressed as percent adhesion. To assess the role of integrins in adhesion to ECs or PCs, isolated neutrophils were pre-incubated with anti-human Mac-1 (clone R15.7, Abcam, Cambridge, MA), anti-human LFA-1 (clone HI111, BioLegend, San Diego, CA) or anti-human β_2_ integrin (anti-human CD18, BioLegend, San Diego, CA) mouse antibodies for 20 min on ice prior to the adhesion assay.

### Neutrophil Motility, Polarization, Velocity, and Movement

ECs or PCs were cultured on gelatin-coated 25 mm circular coverslips until confluent and activated with IL-1β for 4 hr. Neutrophils (at a concentration of 1×10^5^ cells/ml) were then injected into a Sykes-Moore adhesion chamber containing the EC or PC monolayers. After allowing the solution to settle, a time-lapse sequence was captured using a Nikon Eclipse TE2000-U microscope and NIS-Elements imaging software. Images were taken at 15-second intervals over 15 minutes for a total of 61 images and were analyzed using ImageJ software (NIH). Neutrophils were tracked using the Manual Tracking plug-in; results were exported to Excel. Trajectories were plotted using X,Y-coordinates and average velocity was calculated for each neutrophil. Neutrophil motility was calculated by evaluating the number of cells in each video that moved at least 10 µm from the origin. Neutrophil polarization was calculated by evaluating the number of cells in each video extending a pseudopod 3–5 µm in length. For each image sequence, up to ten neutrophils were selected for analysis, with 3 sequences recorded for each volunteer and a minimum of 3 volunteers used for each condition. Cell trajectories shown are a representative selection.

### Neutrophil Transmigration

For transmigration studies, EC monolayers, PC monolayers or EC/PC bilayers were cultured on 3.0 µm pore polycarbonate Transwells (Corning, Corning, NY). To generate EC/PC bilayers, PCs were seeded on the underside of a Transwell membrane and allowed to attach. Following PC attachment, ECs were seeded onto the top side of the Transwell membrane and the cells were cultured together until both monolayers were confluent. Mono- or bilayers were activated for 4 or 24 hr with IL-1β as indicated. Following activation, inserts were washed with HBSS and transferred to six-well plates coated with a thin layer of 1% agarose, which facilitated the removal of transmigrated neutrophils for further analysis and minimized additional activation by the plastic wells. Approximately 3 million freshly isolated (naive) neutrophils from healthy volunteers were placed on top of the insert (ECs facing upward in the case of bilayers) and allowed to transmigrate through the activated monolayers or bilayers for 1 hr. Transmigrated neutrophils were collected from the bottom of the insert and counted by hemacytomer. To further assess the role of ICAM-1 ligands, transmigration studies were conducted using PC monolayers or EC/PC bilayers constructed with ICAM-1-transduced PCs. The role of CD18 integrins on the neutrophil was assessed by pre-incubating isolated neutrophils with anti-β_2_/CD18 (BioLegend) antibody or isotype control antibody for 20 min on ice prior to the transmigration assay. To evaluate the role of neutrophil priming by EC, neutrophils were collected following a 1 hr migration across an EC monolayer then counted, seeded onto PC monolayers and allowed to transmigrate for 1 hr (Post-TEM PC).

### β_2_ Integrin Expression on Neutrophils

For Flow Cytometry analyses, naive neutrophils, IL-8 stimulated, and neutrophils collected following transmigration across EC monolayers were analyzed for β_2_ integrin expression. To do so, neutrophils were labeled with primary anti-human β_2_/CD18 antibody (BioLegend) and anti-mouse IgG-FITC Fab fragment secondary antibody (Sigma-Aldrich) concurrently for 20 minutes, rinsed in PBS, and analyzed using a BD LSRII flow cytometer. As negative controls, both naive neutrophils and TEM neutrophils were incubated with control IgG for 20 minutes.

### Statistical Analysis

Data are expressed as mean ± SE. A minimum of 3 individuals were used as donors for each experiment. Data sets were analyzed for significance by one-way ANOVA with a Tukey test for posthoc analysis and student-T test were performed on paired comparisons; significance was defined at P<0.05, indicated by *, **, or #.

## Results

### Neutrophil Adhesion to Cytokine-activated EC or PC Monolayers

**Figure 1:**
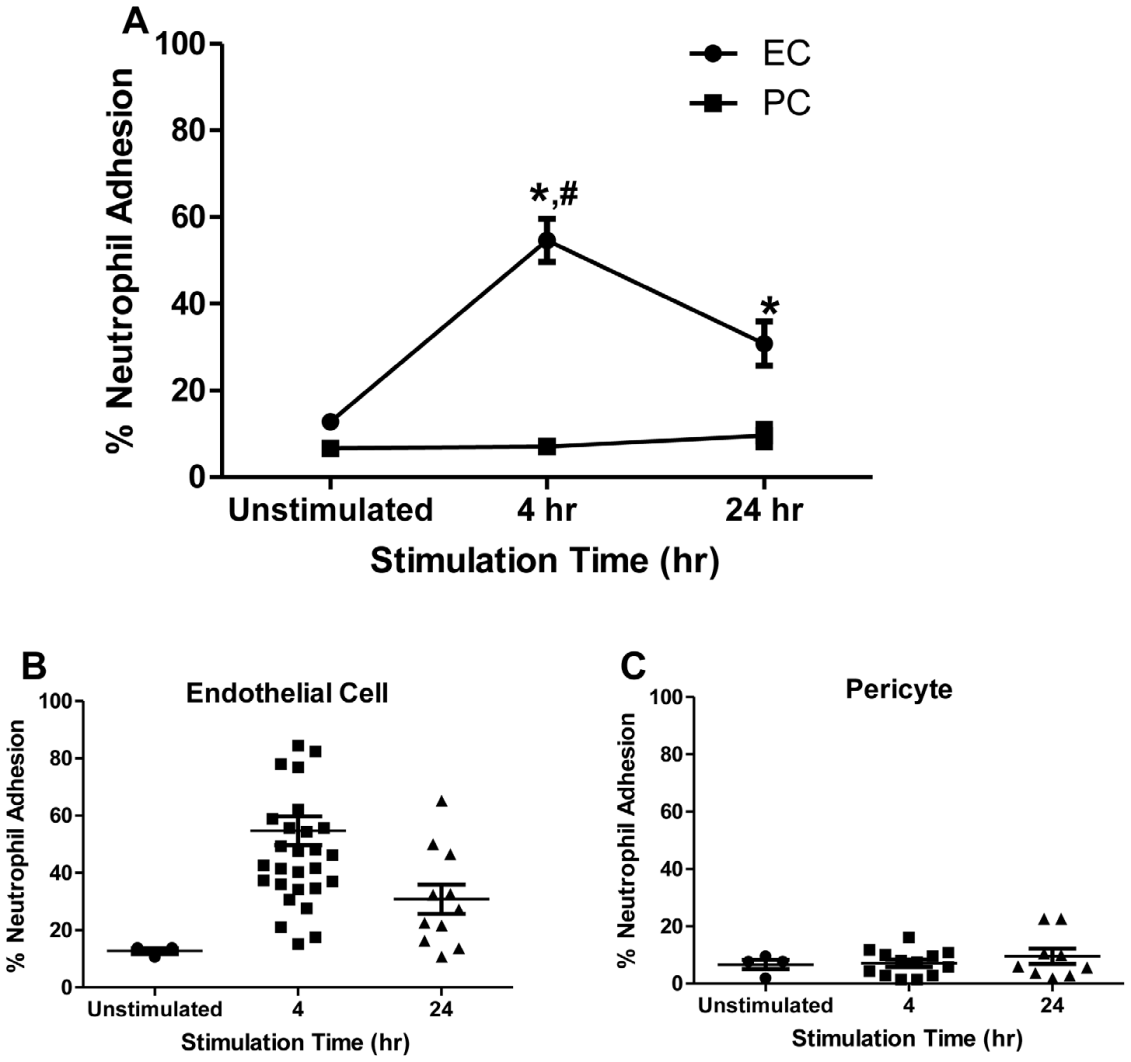
Neutrophil adhesion to IL-1β-activated EC or PC monolayers. Freshly isolated (naïve) neutrophils were seeded onto EC or PC monolayers previously activated with IL-1β for 4 or 24 hr in Sykes-Moore chambers and allowed to adhere prior to counting. Data are represented (A) as a line plot of neutrophil adhesion to EC or PC or as a scatterplot of neutrophil adhesion to (B) EC or (C) PC. Line data represents average neutrophil adhesion ± SEM. *P<0.05, when compared with PC at same timepoints; ^#^P<0.05 when compared with unstimulated EC.

A necessary precursor to neutrophil entry into peripheral tissues is neutrophil adhesion to the ECs that line the lumen of microvessels, predominantly in the post-capillary venules. This process has been most often studied in vitro using monolayers of human ECs isolated from umbilical veins. Here we have compared the interactions of neutrophils with human umbilical vein ECs with interactions of human Thy-1^+^ NG2^+^ PCs isolated from placental microvessels. We first examined neutrophil adhesion to EC or PC monolayers under either basal culture conditions or following activation by IL-1β, a cytokine known to stimulate neutrophil adhesion to and transmigration through ECs, for 4 or 24 hr. Neutrophil adhesion to unactivated PC and EC monolayers was minimal (Fig. 1). As expected, EC monolayers activated for 4 hr with IL-1β exhibited high levels of neutrophil adhesion; the level of adhesion remained well above that of controls but somewhat declined at later timepoints of IL-1β pre-treatment. Neutrophil adhesion remained low on IL-1β activated PC across all timepoints. Thus, neutrophil adhesion to cytokine-activated ECs is significantly more robust than adhesion to activated PCs.

### Neutrophil Motility, Polarization, Velocity, Distance from Origin and Movement on Cytokine Activated EC or PC Monolayers

**Figure 2:**
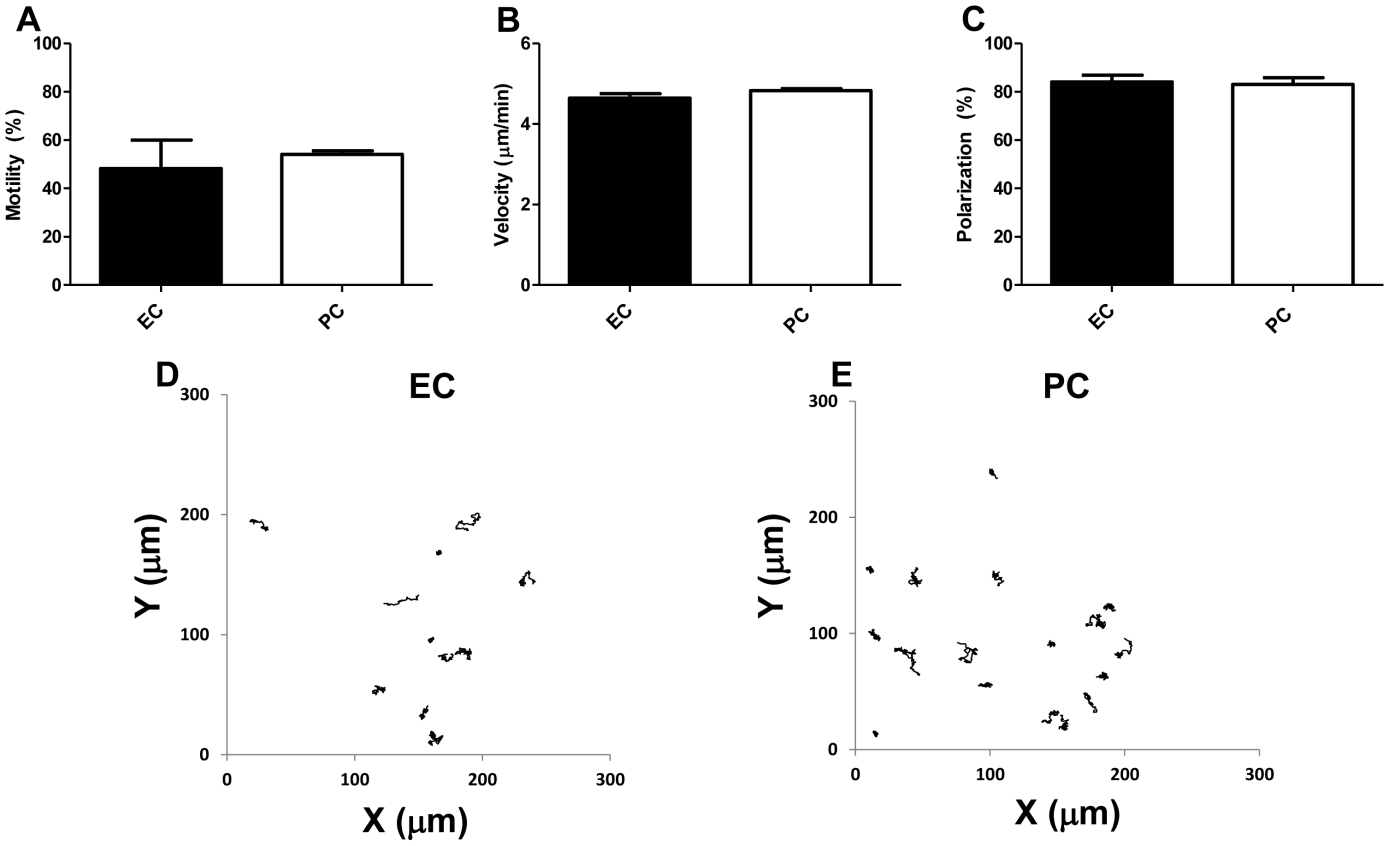
Neutrophil motility, polarization, and velocity. Freshly isolated neutrophils were seeded onto EC or PC monolayers (activated for 4 hr with IL-1β) in Sykes-Moore chambers and neutrophil (A) motility, (B) polarization, (C) velocity, (D) movement on EC and (E) movement on PC was time-lapse captured for 15 min. Bars represent average motility/polarization/velocity ± SEM.

**Figure 3:**
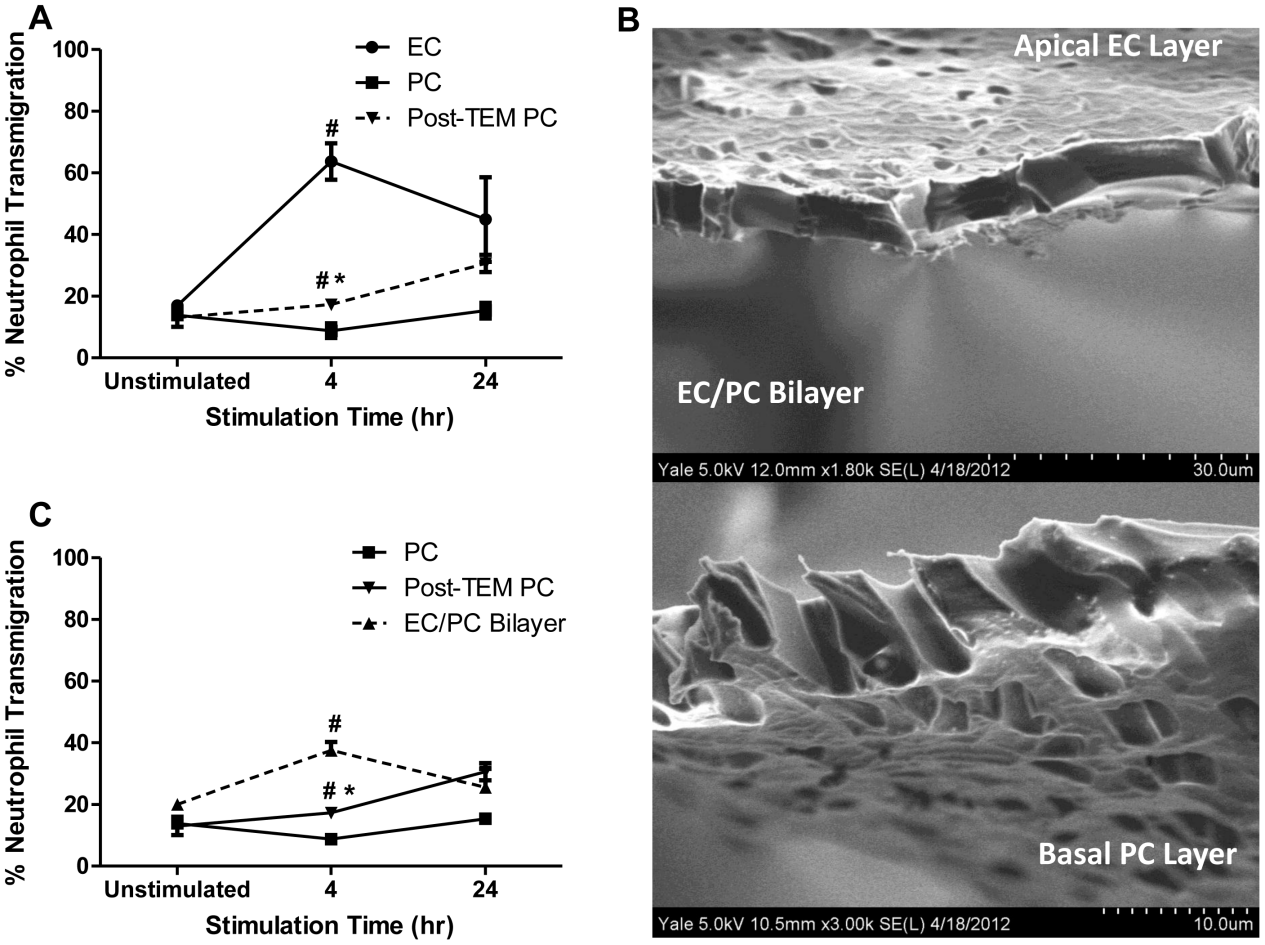
Neutrophil transmigration across IL-1β activated EC or PC monolayers. Freshly isolated neutrophils were seeded onto monolayers (activated with IL-1β for 4 or 24 hr) and allowed to transmigrate for 1 hr. Data are represented as a (A) line plot of naive neutrophil transmigration across EC or PC, and transendothelial migrated neutrophils (Post-TEM) transmigration across PC. Figure 3B is demonstrative of (B) EC and PC co-cultured on a single membrane to form an EC/PC bilayer. (C) Freshly isolated naive neutrophils were seeded onto EC/PC bilayers (activated with IL-1β for 4 or 24 hr) and allowed to transmigrate for 1 hr. Line data represent average neutrophil transmigration ± SEM. ^#^P<0.05, when compared to 4 hr activated PC, *P<0.05, when compared to 4 hr activated EC or EC/PC Bilayer.

Neutrophils adherent to the venular EC surface *in vivo* become activated by EC-bound chemokines and then crawl to reach the vicinity of an inter-EC junction. Crawling is an important precursor to transendothelial migration and is largely dependent upon neutrophil Mac-1 interaction with EC-expressed ICAM-1 [26]. We compared the motility (48.2 and 54.1%, respectively, polarization (84.2 and 83.1%, respectively), and velocity (4.6 and 4.8 µm/min, respectively) of adherent neutrophils on 4 hr IL-1β-activated EC and PC monolayers. Despite profound differences in the absolute number of adherent neutrophils between the two types of cell monolayers (Fig. 2 and 3), we found no significant differences between the capacities of IL-1β-activated EC and PC monolayers to support overall motility of those neutrophils that are adherent (Fig. 2A–E).

### Neutrophil Transmigration Across Cytokine-activated EC or PC Monolayers

To compare the capacities of EC and PC monolayers act to support diapedesis into tissues, we next examined neutrophil transmigration across IL-1β-activated (for 4 or 24 hr) EC or PC monolayers. IL-1β-treated ECs supported higher levels of neutrophil transmigration early after activation; neutrophil transmigration was highest following 4 hr activation, subsequently decreasing at 24 hr of activation (Fig. 3A). Neutrophil transmigration across IL-1β-activated PC monolayers was comparatively low at all timepoints of cytokine treatment, but exhibited a slight increase after 24 hr of activation versus an unactivated control or after 4 hr of cytokine treatment (Fig. 3A). Neutrophil transpericyte migration could be significantly enhanced when neutrophils were allowed to transmigrate an IL-1β activated EC layer (post-TEM) before exposure to the IL-1β activated PC layer (Figure 3A). Overall, these data suggest that transmigration of naive (non-transmigrated) neutrophils is much more robustly supported by activated EC monolayers than activated PC monolayers. However, the act of transendothelial migration appears to prime neutrophils for an efficient encounter with subendothelial pericytes.

To more fully model the venular wall, which is formed by ECs supported by subendothelial PC, we developed an EC/PC bilayer (Fig. 3B) and used it to evaluate the ability of neutrophils to transmigrate in either its quiescent and IL-1β activated state. In this manner, neutrophils will undergo transendothelial migration prior to encountering the subendothelial PC. Unactivated bilayers support a low level of neutrophil transmigration, comparable to that supported by either unactivated EC or PC monolayers. In contrast, bilayers activated by IL-1β for 4 hours supported a level of transmigration intermediate to those of activated EC and PC monolayers (Fig. 3C). However, the level of transbilayer migration was enhanced, compared to that of post-TEM neutrophil transmigration across PC, suggesting that EC are necessary for high levels of neutrophil recruitment and capture through the microvascular PC layer, as PC are unable to recruit these cells independently. Additionally, these data suggest that the composite microvascular structure retains an inhibitory contribution from PCs that limit, but do not prevent neutrophil diapedesis following IL-1β activation.

### IL-8 and ICAM-1 Expression on Cytokine Activated EC or PC Monolayers

**Figure 4:**
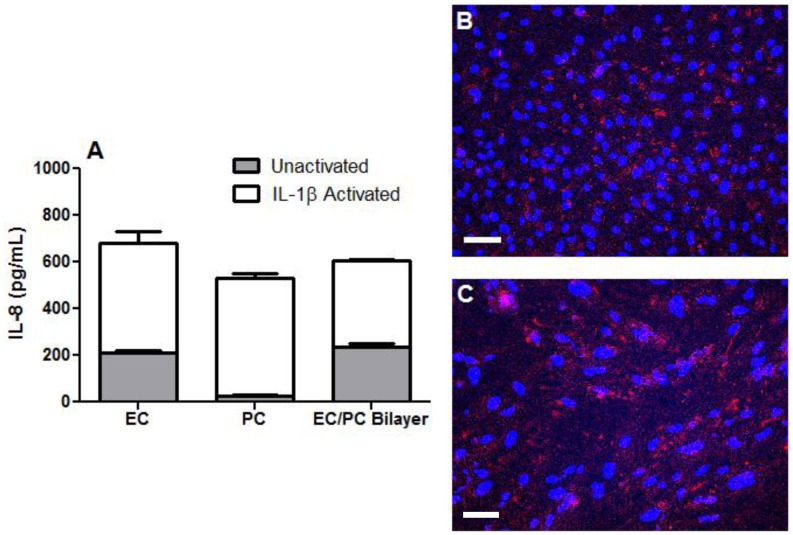
IL-8 expression and presentation. (A) EC or PC monolayers and EC/PC bilayers were grown until confluence on Transwells and incubated for 4 hr with IL-1β. Following incubation, media were removed and analyzed for IL-8 by ELISA. Bars represent average IL-8 expression ± SEM. IL-1β activated (B) EC and (C) PC monolayers were stained and imaged for cell surface bound IL-8. Bar in B and C 100 µm.

The large differences demonstrated in neutrophil adhesion to and transmigration across EC and PC monolayers could be due to differences in surface chemokine expression and/or LAM expression. We therefore examined if differences in IL-8 expression and/or presentation were responsible for the differences we have observed between neutrophil interactions with EC and PC monolayers. The overall expression of IL-8 measured in conditioned media from IL-1β activated cultures was similar among EC and PC monolayers and EC/PC bilayers (Fig. 4A). Immunostaining of cell surface-bound IL-8 on EC and PC monolayers demonstrated no discernible difference between surface display of IL-8 on ECs vs. PCs; IL-8 was well distributed across the surface of the both cell monolayers (Fig. 4B,C). These results suggest that the lower levels of neutrophil adhesion to and transmigration across PC monolayers were not likely to be a result of inadequate IL-8 production or its cell surface display.

**Figure 5:**
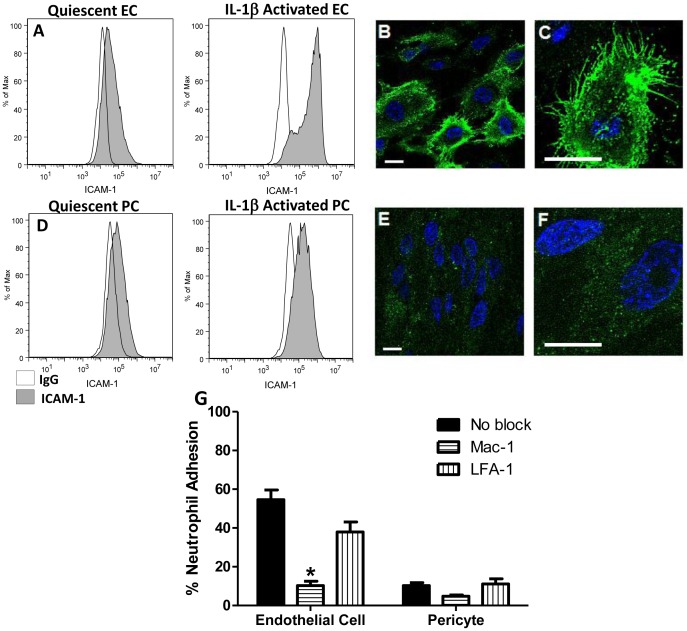
EC and PC ICAM-1 expression and effects of CD18 blocking on neutrophil adhesion. ICAM-1 expression on (A) EC or (D) PC monolayers before and after 4 hr IL-1β activation was assessed by flow cytometry (IgG labeled control white). ICAM-1 expression on (B,C) EC and (E,F) PC following 4 hr IL-1β activation was imaged using confocal microscopy. Scale bars are 20 µm. (G) Neutrophil adhesion inhibition to IL-1β activated EC or PC monolayers. Freshly isolated neutrophils were pre-incubated with anti-Mac-1 or anti-LFA-1 antibodies and seeded onto EC or PC monolayers (activated for 4 with IL-1β) in Sykes-Moore chambers and allowed to adhere prior to counting. Bars represent average neutrophil adhesion ± SEM. *P<0.05, when compared to the no block control.

ICAM-1 is the major LAM responsible for neutrophil adhesion to cytokine-activated ECs [30]. Activation of ECs by IL-1β (4 hr) resulted in levels ICAM-1 surface expression (Fig. 5A) that are significantly higher than ICAM-1 surface expression on IL-1β treated PCs (Fig. 5D), as assessed by flow cytometry analysis. Confocal immunofluorescence microscopy of ICAM-1 expression on EC and PC monolayers revealed that EC monolayers displayed broad ICAM-1 distribution over each cell surface in conjunction with pronounced localized expression around the lateral borders of the cell with a further concentration on numerous stellate projections (Fig. 5B, C). In contrast, PC monolayers exhibited a more evenly distributed distribution of ICAM-1, albeit at significantly lower quantities compared to EC, and did not demonstrate increased display of ICAM-1 at the lateral borders or associated with stellate projections (Fig. 5E, F). The significantly higher ICAM-1 expression exhibited on EC monolayers and its concentration at EC lateral borders is consistent with the hypothesis that ICAM-1 expression and localization may serve to mediate higher levels of neutrophil adhesion and transmigration, respectively on IL-1β-activated PC monolayers.

**Figure 6:**
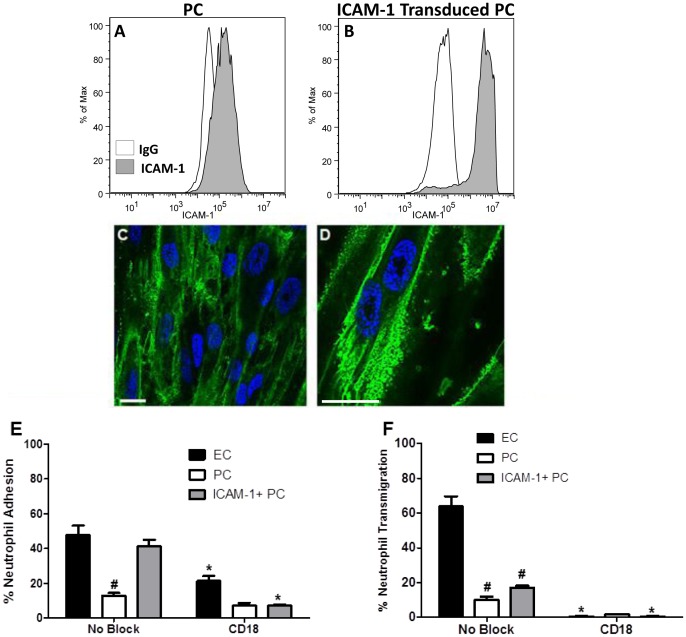
ICAM-1 transduced PC. ICAM-1 expression on (A) non-transduced PC and (B) ICAM-1-transduced PC (ICAM-1+PC) monolayers following 4 hr of activation by IL-1β. ICAM-1 assessed using flow cytometry. ICAM-1 expression on (C,D) ICAM-1 transduced PC following 4 hr of IL-1β activation imaged using confocal microscopy. Bar in (C,D), 20 µm.(E) Neutrophil adhesion on EC, PC or ICAM-1 transduced PC monolayers following 4 hr of IL-1β activation. Freshly isolated neutrophils were pre-incubated with anti-CD18 antibodies and seeded onto the monolayers in Sykes-Moore chambers and allowed to adhere prior to counting. Bars represent average neutrophil adhesion ± SEM. ^#^P<0.05, when compared to no block EC control. *P<0.05, when compared to no block of the same cell control. (F) Neutrophil transmigration across EC, PC or ICAM-1 transduced PC monolayers following 4 hr IL-1β activation. Freshly isolated neutrophils were pre-incubated with anti-CD18 antibodies and seeded onto the Transwell monolayers and allowed to transmigrate for 1 hr. Bars represent average neutrophil transmigration ± SEM. ^#^P<0.05, when compared to no block EC control. *P<0.05, when compared to no block of the same cell layer.

We took two approaches to determine whether these observed differences in ICAM-1 expression levels between activated ECs and PCs can explain differences in neutrophil adhesion to and/or transmigration through EC or PC monolayers in our experiments. First, neutrophils were incubated with anti-Mac-1 (anti-α_M_β_2,_ anti-CD11b/CD18) or anti-LFA-1 (anti- α_L_β_2_, anti-CD11a/CD18) antibody, neutralizing the principal receptors for ICAM-1, before being seeded onto IL-1β-activated (for 4 hr) EC or PC monolayers. Neutrophil pre-incubation with an anti-Mac-1 antibody resulted in significant inhibition of adhesion to IL-1β-activated EC monolayers. We also observed a slight reduction in the already low levels of neutrophil adhesion to IL-1β activated PC monolayers (Fig. 5G). Antibodies against LFA-1 were less effective as inhibitors of neutrophil adhesion to either EC or PC. Second, we retrovirally transduced PCs to express higher levels of ICAM-1. ICAM-1 expression on unactivated and IL-1β-activated transduced PCs (ICAM-1+PC) was measured using flow cytometry to verify increased adhesion molecule expression on the cell surface compared to IL-1β activated non-transduced PC (Fig. 6A, B). Flow cytometry verified no measurable increase in ICAM-1 expression between unactivated and IL-1β activated ICAM-1 transduced PC (data not shown). Confocal fluorescence microscopic images of ICAM-1 expression on IL-1β-activated ICAM-1-transduced PC monolayers demonstrated high levels of ICAM-1 expression (Fig. 6C, D), similar to that of EC (Fig. 5B, C) and higher than those on non-transduced PC (Fig. 5E, F). Transduced ICAM-1 appeared well distributed across the cell surface and now displayed additional localization around the outer edges of the cell; however, ICAM-1 transduced PCs did not display ICAM-1-rich projections like those seen on the EC monolayers. Importantly, transduction of PCs with ICAM-1 did not alter expression or distribution of IL-1β-induced IL-8 when compared to non-transduced PC (Fig. 4C). The effect of ICAM-1 transduction of PCs in the absence of IL-1β significantly increased the capacity of these cells to bind neutrophils, as unactivated ICAM-1 transduced PC were able to support 34.1% neutrophil adhesion, as compared to 6.6% on unactivated PC. However, when ICAM-1-transduced PC monolayers were activated with IL-1β for 4 hr, they exhibited slightly higher rates of neutrophil adhesion (41.4%) and supported similar levels of adhesion as cytokine-activated ECs (Fig. 6E). Neutrophil pre-incubation with an anti-Mac-1 integrin blocking antibody resulted in a decrease in adhesion to IL-1β-activated ICAM-1-transduced PCs as previously noted for adhesion to cytokine-activated ECs, verifying that transduced PC were presenting increased amounts of functional ICAM-1 to mediate the increase in neutrophil adhesion via Mac-1 binding. These results suggest that the large differences exhibited in neutrophil adhesion to PC and EC monolayers may be attributed to the low levels of ICAM-1 expression on non-transduced PC.

Having observed increased neutrophil adhesion by ICAM-1 transduction of PCs we proceeded to examine whether the increased ICAM-1expression on PCs would similarly increase their competence to support neutrophil transmigration. Unactivated ICAM-1-transduced PC were able to support 14.2% neutrophil transmigration, no different from unactivated, non-transduced PC (13.9%). Despite IL-1β activation for 4 hr, ICAM-1-transduced PC monolayers still did not support significantly greater levels of transmigration (17.0%) and still showed much lower rates of transmigration compared to cytokine-activated ECs (63.7%) (Fig. 6F). However, neutrophils pre-incubated with an anti-CD18 (β_2_ integrin) blocking antibody did result in a decrease in transmigration across IL-1β activated EC, PC or ICAM-1-transduced PC monolayers, consistent with a necessary role for ICAM-1 expression in PC- as well as EC-supported transmigration. However, unlike adhesion, increased ICAM-1 expression is not sufficient to support higher levels of transmigration through a cytokine-activated PC monolayer, implying a role for other required processes or interactions.

### EC-primed Neutrophil CD18-dependent Transmigration through PC

**Figure 7:**
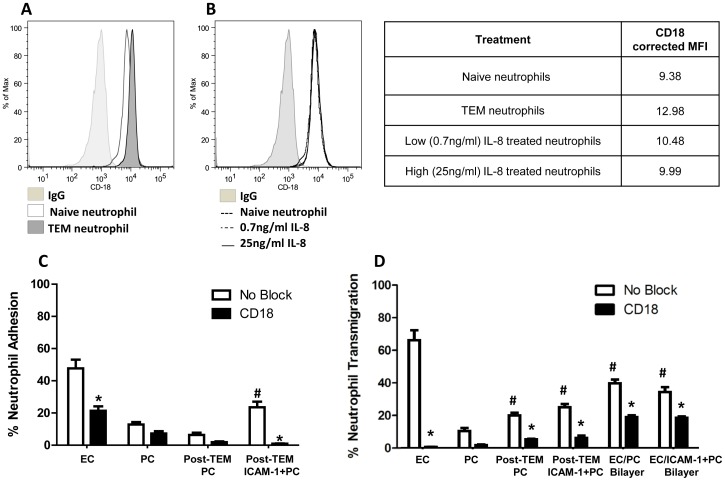
CD18-dependent post-transendothelial migration. (A) CD18 (β_2_ integrin) expression on naïve and transendothelial migrated (TEM) neutrophils (across 4 hr IL-1β activated EC monolayers) and (B) naive 0.7 ng/ml IL-8 and 25 ng/ml IL-8 stimulated neutrophils assessed by flow cytometry. Table 1. Corrected mean fluorescent intensity (MFI) reflect CD18 expression from flow cytometry. (C) Neutrophil adhesion on EC, PC, post-TEM PC, or post-TEM ICAM-1+PC monolayers following 4 hr IL-1β activation. Freshly isolated neutrophils were pre-incubated with anti-CD18 antibodies and seeded onto the monolayers in Sykes-Moore chambers and allowed to adhere prior to counting. Bars represent average neutrophil adhesion ± SEM. *P<0.05, when compared to no block EC control. (D) Neutrophil transmigration across EC, PC, post-TEM PC or post-TEM ICAM-1+PC monolayers, and EC/PC bilayer or EC/ICAM-1+ PC bilayer following 4 hr IL-1β activation. Freshly isolated neutrophils were pre-incubated with anti-CD18 antibodies and seeded onto the Transwell monolayers and allowed to transmigrate for 1 hr. Bars represent average neutrophil transmigration ± SEM. *P<0.05, when compared to no block control of the same cell layer(s). ^#^P<0.05, when compared to no block EC and PC control.

In a final series of experiments we examined the influence of neutrophil ‘priming’ by the ECs on neutrophil interactions with PCs by analyzing adhesion to and transmigration through PC monolayers following transmigration through cytokine-activated EC monolayers (post-TEM neutrophils). As previously reported, the act of transendothelial migration alters the phenotype of the neutrophil by increasing the expression of CD18 integrins on the neutrophil surface (Fig. 7; Table 1) [1], [16]. This phenotypic change in neutrophil integrin expression appears to require interaction with ECs as neutrophil incubation with IL-8 alone does not result in increased CD18 expression (Fig. 7A, B). Post-TEM neutrophils did not exhibit a significantly higher level of adhesion to cytokine-activated PC unless the PC were ICAM-1-transduced (Fig. 7C). However, post-TEM neutrophils did exhibit higher levels of transmigration across both cytokine-activated PC monolayers and ICAM-1-transduced cytokine-activated PC monolayers when compared to naïve neutrophils (Fig. 7D). As a second method of evaluating EC-primed neutrophil transpericyte migration, we utilized cytokine-activated EC/PC bilayers. Consistent with the effect of EC monolayers upon the behavior of neutrophils, levels of transmigration across EC/PC bilayers was mid-way between that of neutrophil transmigration across EC monolayers (high) and PC monolayers (low). Bilayers constructed with ICAM-1-transduced PCs (EC/ICAM-1+ PC bilayers) supported transmigration at levels similar to that of a control cytokine-activated EC/PC bilayer. In other words, increased ICAM-1 expression on the PCs had no further effect on diapedesis across EC/PC bilayers, though it effectively increases the ability of leukocytes to adhere to pericytes. To further evaluate the role of ICAM-1 in this system, we examined the effect of blocking CD18 integrin interactions with their ligands using monoclonal antibody. Analysis of the mean percent reduction in transmigration following anti-CD18 treatment of freshly isolated neutrophils indicates that EC transmigration is reduced by 99.1% while PC transmigration is reduced by 83.7%. Post-TEM neutrophil transmigration through PC is reduced by 73.7%, with post-TEM ICAM-1-transduced PC transmigration reduced by 75.6%. However, neutrophil CD18 blocking reduced transmigration across EC/PC bilayers and EC/ICAM-1+PC bilayers by only 52.5% and 46.1%, respectively. Together, these results indicate that the act of transmigration across the EC layer reduces the dependence of transpericyte migration upon CD18 ligand ICAM-1.

**Table 1:** Corrected mean fluorescent intensity (MFI) of CD18 expression on neutrophils.

Treatment	CD18 corrected MFI
Naive neutrophils	9.38
TEM neutrophils	12.98
Low (0.7 ng/ml) IL-8 treated neutrophils	10.48
High (25 ng/ml) IL-8 treated neutrophils	9.99

## Discussion

The use of human EC cultures to analyze the process of neutrophil recruitment from blood into tissue has been highly successful and many of the molecules identified by such models have been confirmed to be physiologically relevant in living rodents both by antibody blocking studies and by genetic mutational analyses [1], [32]. The vast majority of leukocyte extravasation from the blood into infected tissue occurs through post-capillary venules where the EC lining of the vessel is invested by a network of PCs embedded within a shared basement membrane; thus neutrophils must actually diapedese through two distinct cell layers, EC and PC, in order to reach perivascular tissue [33], [34]. The contribution of the PC cell layer to neutrophil transmigration and the leukocyte adhesion cascade has, until recently, been ignored [4]–[7], [34]. Furthermore, while most *in vitro* models have used human neutrophils and human ECs, the few investigations that have considered PCs have largely used PCs from non-human sources, raising the concern that crucial interactions may have been missed because of species incompatibilities of receptors and their ligands. For example, human leukocyte integrins poorly interact with rodent ICAM-1. Here we describe the use of an *in vitro* system consisting entirely of human cells to examine the regulation of neutrophil activity by IL-1β activated EC and PC monolayers. We used our system to investigate three phenomena: 1) The ability of EC and PC to differentially support neutrophil adhesion, locomotion and subsequent transmigration, 2) potential contributions of PC to composite microvascular support of neutrophil tissue infiltration and 3) the extent to which these events are ICAM-1/Mac-1 dependent. We identify a crucial role for recognition of ICAM-1 in static adhesion to and a necessary but insufficient role for ICAM-1 in transmigration through cytokine-activated monolayers of both cell types using freshly isolated neutrophils. Our studies demonstrate that interactions with PCs are limited by their lesser expression of this protein. Most importantly, we show that the process of transendothelial migration primes neutrophils both for ICAM-1 recognition and for efficient transpericyte migration, a process that is, at least in part, ICAM-1-independent involving an as yet unidentified receptor/counter-receptor pair(s). Our observations suggest that the combination of EC and PC presented simultaneously in a microvascular simulating bilayer will provide signals to one another, and to the neutrophil that are unique to the bilayer system, not experienced by neutrophils interacting with either EC or PC monolayer individually. The novel EC/PC co-culture bilayer system we have developed may provide a means by which more comprehensive descriptions of neutrophil diapedesis can be investigated.

A primary conclusion of this study is that cytokine-activated ECs facilitate the diapedesis of freshly isolated neutrophils while PCs do not (Fig. 1 & 3). We have broken up this process into several steps and show that PCs are specifically defective at supporting adhesion and transmigration but can adequately support crawling of neutrophils on the cell surface. ICAM-1 is largely responsible for neutrophil firm adhesion to the endothelium and is involved in creation of an apical “cup-like” structure providing neutrophils with directional guidance towards preferential (ICAM-1-rich, laminin-low) regions for transmigration [1], [2]. Surprisingly, PCs simply do not express sufficient ICAM-1 to support these processes yet still support crawling of a subset of neutrophils that do attach, suggesting that ICAM-1 localization is key to neutrophil transmigration events. Confocal immunofluorescence microscopy of ICAM-1 expression on EC and PC monolayers confirmed these differences in ICAM-1 expression and further revealed striking differences in ICAM-1 distribution and localization on ECs compared to PCs. Transduction of PCs to overexpress ICAM-1 corrects the deficit in adhesion but not in transmigration. Because the ICAM-1/Mac-1 interaction is just one, albeit an important interaction, among the many implicated in neutrophil transendothelial migration, it is not surprising that an increase in ICAM-1 expression on the PC alone did not fully support neutrophil transmigration. The deficit both in adhesion to and in transmigration across PC monolayers is partially remedied by EC-mediated priming of the neutrophil as it migrates across the EC monolayer. Whether ICAM-1 is simply acting as a guide or fully arbitrating transmigration has yet to be delineated [2]. Our results indicate that PCs naturally express little ICAM-1 even after cytokine activation and do not concentrate it at their lateral borders where gaps are likely to occur. They do, however, produce and display quantities of IL-8 comparable to that made by ECs. IL-8 is the major chemokine produced by IL-1β-activated human ECs that stimulates transendothelial migration of bound neutrophils [27]–[29]. The more relevant contributions of IL-8 to adhesion and transmigration may come from surface displayed chemokine rather than secreted soluble chemokine. We identify no great difference in the production or presentation of IL-8 between EC or PC activated with IL-1β.Other differences between ECs and PCs may also be important. Neutrophil transmigration across an EC monolayer is dependent upon both PECAM-1 (CD31) and CD99 expressed on both cell types. Maier *et al* demonstrated a lack of PECAM-1 on PCs [35], and while the expression of CD99 protein has not yet been examined, PCs do express high levels of mRNA encoding this protein. Interestingly, Bixel *et al* demonstrated that blocking CD99 antibodies effectively stopped neutrophil transvascular migration at the perivascular level of the mouse cremaster muscle; neutrophils were trapped in the basement membrane along the pericyte layer [36], [37]. It is likely that alternative ligands are available on the PC at their lateral edge that can be recognized by receptors, other than Mac-1, that are upregulated on the neutrophil during transmigration across an EC monolayer.

Recent *in vivo* studies of leukocyte extravasation through microvasculature of the mouse cremaster muscle have demonstrated that neutrophils primarily cross PC layers through large PC gaps with cell borders containing low levels of laminin and type IV collagen, and high levels of ICAM-1 and CXCL-1, a mouse equivalent to IL-8 [6]. Our system utilizes only human cells and does not provide large PC gaps for neutrophil transmigration, but rather presents a truly confluent monolayer of PC that models the highly PC-invested post-capillary venule of the lung, eye and skin. This considerable difference in model, both between species and physiological location (pulmonary post-capillary venule versus the cremaster muscle), may explain a difference in the frequency of neutrophil transmigration through the pericyte layer.

Neutrophil transmigration across the EC/PC bilayer resulted in rates of transmigration midway between that of the EC and PC monolayers, further validating the specific roles of the EC and PC layers and further indicating that transendothelial migration alters the neutrophils. The suggestion is that additional ligands, recognition of which is enhanced by neutrophil transendothelial migration, play a role in transmigration across PCs. Furthermore, the changes observed in post-TEM neutrophil behaviors are more apparent in experiments using EC/PC bilayers than in those involving sequential transmigration across EC and PC monolayers, suggesting either that some relevant changes may be short-lived or that the properties of the vascular cells in bilayers differ from those of the same cells in monolayer cultures. The phenomena of neutrophil “priming” by transmigration across activated EC layers is notably relevant to transpericyte migration because of the differential regulation of multiple neutrophil integrins (α_6_β_1_, α_3_β_1_, α_4_β_1_, α_5_β_1_, α_9_β_1_, α_2_β_1_, α_M_β_2_) [16], [31]. The increase in neutrophil transmigration across the EC/PC bilayer compared to the PC monolayer may be attributed, in part, to the ICAM-1/Mac-1 (α_M_β_2_) binding combination, as Mac-1 expression increases following transendothelial migration (Fig. 7) and CD18 antibodies effectively inhibited neutrophil transmigration across EC/PC bilayers (Fig. 7D). Our observation using inhibitory antibody reveals that Mac-1 is not fully responsible for transpericyte migration; rather, changes in alternative neutrophil adhesion molecule expression may contribute in large part to this process (Fig. 7D). Some of these changes may be short-lived as the Mac-1-independent effects appear to be more pronounced in experiments using EC/PC bilayers than those using sequential EC and PC monolayers. Alternatively, the bilayer system may change the ECs and/or PCs in a manner that alters their effects upon and/or interactions with neutrophils.

In summary, our new functional assays allowed us to evaluate how EC and PC monolayers differentially regulate and initiate events leading to neutrophil transmigration. Here we analyzed the separate roles of the EC and PC as well as how they function together to mediate neutrophil transmigration. Our focus in the present study has been on the effects that PC may exert on neutrophil transmigration, heretofore largely investigated *in vitro* using EC monolayers. Historically, PC effects on EC have focused upon control of proliferation and upon vascular integrity and leakiness. Although it may seem evident that neutrophil transendothelial migration would affect permeability, the surprising finding is that it does not [38]. It will be interesting to observe if the presence of PC in bilayer cultures will exert on effect on basal permeability or create conditions under which neutrophils may cause increased leak. Very little is known about PC signaling and the influence this cell layer has on the endothelium. In future studies, we hope to further delineate the various contributions of the PC layer by examining the specific molecular influence of PC paracrine signaling, PC protein deposition and PC/EC cell contact on the ability of PC to alternatively alter the EC ability to support neutrophil recruitment. In addition, inflammatory chemokines can regulate adhesion molecule and cytokines to promote juxtacrine signals between two cell types. Clinical potential for this research is broad and far-reaching, including elucidation of therapeutic targets against inflammatory diseases that manifest in the lung, eye and skin, where PC are present in high numbers [14]. In injured tissues and vascular models, targets such as cytokine presenting glycoproteins or adhesion molecules would be ideal targets for therapeutic purposes [39]. Finally, taken in the broader context of literature relating microvasculature to regulation of inflammation and leukocyte recruitment, we have presented a novel model utilizing human cells for further investigation of the multiple components that contribute to the events in the leukocyte adhesion cascade and subsequently, inflammation. Our model combines the inclusion of the PC layer; while these cells have yet to be widely studied *in vitro,* a method to successfully isolate, grow and characterize a pure culture of these cells was developed by Maier *et al* in 2010 [12]. The PC cultures used in this study were isolated and grown according to this protocol and were characterized for the appropriate PC markers. The use of the PC combined with a complex three human cell system creates a unique model that, while taken from a reductionist viewpoint, allows us to systematically address the contributions and behavior of each cell type in order to better assess the inflammatory response as a whole.

## References

[1] Woodfin A, Voisin MB, Nourshargh S (2010) Recent developments and complexities in neutrophil transmigration. Curr Opin Hematol 17: 9–17. 19864945 10.1097/MOH.0b013e3283333930 PMC2882030

[2] Carman CV, Springer TA (2004) A transmigratory cup in leukocyte diapedesis both through individual vascular endothelial cells and between them. J Cell Biol 167: 377–388. 15504916 10.1083/jcb.200404129 PMC2172560

[3] Muller WA (2011) Mechanisms of leukocyte transendothelial migration. Annu Rev Pathol 6: 323–344. 21073340 10.1146/annurev-pathol-011110-130224 PMC3628537

[4] Wang S, Voisin MB, Larbi KY, Dangerfield J, Scheiermann C, et al (2006) Venular basement membranes contain specific matrix protein low expression regions that act as exit points for emigrating neutrophils. J Exp Med 203: 1519–1532. 16754715 10.1084/jem.20051210 PMC2118318

[5] Voisin MB, Pröbstl D, Nourshargh S (2010) Venular basement membranes ubiquitously express matrix protein low-expression regions: characterization in multiple tissues and remodeling during inflammation. Am J Pathol 176: 482–495. 20008148 10.2353/ajpath.2010.090510 PMC2797906

[6] Proebstl D, Voisin MB, Woodfin A, Whiteford J, D’Acquisto F, et al (2012) Pericytes support neutrophil subendothelial cell crawling and breaching of venular walls in vivo. J Exp Med 209: 1219–1234. 22615129 10.1084/jem.20111622 PMC3371725

[7] Stark K, Eckart A, Haidari S, Tirniceriu A, Lorenz M, et al (2013) Capillary and arteriolar pericytes attract innate leukocytes exiting through venules and ‘instruct’ them with pattern-recognition and motility programs. Nat Immunol 14: 41–51. 23179077 10.1038/ni.2477

[8] Shepro D, Morel NM (1993) Pericyte physiology. FASEB J 7: 1031–1038. 8370472 10.1096/fasebj.7.11.8370472

[9] Hirschi KK, D’Amore PA (1996) Pericytes in the microvasculature. Cardiovasc Res 32: 687–698. 8915187

[10] Allt G, Lawrenson JG (2001) Pericytes: cell biology and pathology. Cells Tissues Organs 169: 1–11. 11340256 10.1159/000047855

[11] Orlidge A, D’Amore PA (1987) Inhibition of capillary endothelial cell growth by pericytes and smooth muscle cells. J Cell Biol 105: 1455–1462. 3654761 10.1083/jcb.105.3.1455 PMC2114828

[12] Maier CL, Shepherd BR, Yi T, Pober JS (2010) Explant outgrowth, propagation and characterization of human pericytes. Microcirculation 17: 367–380. 20618694 10.1111/j.1549-8719.2010.00038.x PMC2928225

[13] Helmbold P, Wohlrab J, Marsch WC, Nayak RC (2001) Human dermal pericytes express 3G5 ganglioside–a new approach for microvessel histology in the skin. J Cutan Pathol 28: 206–210. 11426828 10.1034/j.1600-0560.2001.028004206.x

[14] Armulik A, Abramsson A, Betsholtz C (2005) Endothelial/pericyte interactions. Circ Res 97: 512–523. 16166562 10.1161/01.RES.0000182903.16652.d7

[15] Jaffe EA, Nachman RL, Becker CG, Minick CR (1973) Culture of human endothelial cells derived from umbilical veins. Identification by morphologic and immunologic criteria. J Clin Invest 52: 2745–2756. 4355998 10.1172/JCI107470 PMC302542

[16] Gonzalez AL, El-Bjeirami W, West JL, McIntire LV, Smith CW (2007) Transendothelial migration enhances integrin-dependent human neutrophil chemokinesis. J Leukoc Biol 81: 686–695. 17164427 10.1189/jlb.0906553

[17] Gopalan PK, Burns AR, Simon SI, Sparks S, McIntire LV, et al (2000) Preferential sites for stationary adhesion of neutrophils to cytokine-stimulated HUVEC under flow conditions. J Leukoc Biol 68: 47–57. 10914489

[18] Burns AR, Bowden RA, MacDonell SD, Walker DC, Odebunmi TO, et al (2000) Analysis of tight junctions during neutrophil transendothelial migration. J Cell Sci 113 (Pt 1): 45–57. 10.1242/jcs.113.1.45 10591624

[19] Burns AR, Walker DC, Brown ES, Thurmon LT, Bowden RA, et al (1997) Neutrophil transendothelial migration is independent of tight junctions and occurs preferentially at tricellular corners. J Immunol 159: 2893–2903. 9300713

[20] Gimbrone MA, Cotran RS, Folkman J (1974) Human vascular endothelial cells in culture. Growth and DNA synthesis. J Cell Biol 60: 673–684. 4363161 10.1083/jcb.60.3.673 PMC2109230

[21] Thornton SC, Mueller SN, Levine EM (1983) Human endothelial cells: use of heparin in cloning and long-term serial cultivation. Science 222: 623–625. 6635659 10.1126/science.6635659

[22] Maier CL, Shepherd BR, Yi T, Pober JS (2010) Explant outgrowth, propagation and characterization of human pericytes. Microcirculation 17: 367–380. 20618694 10.1111/j.1549-8719.2010.00038.x PMC2928225

[23] Clark PR, Manes TD, Pober JS, Kluger MS (2007) Increased ICAM-1 expression causes endothelial cell leakiness, cytoskeletal reorganization and junctional alterations. J Invest Dermatol 127: 762–774. 17195014 10.1038/sj.jid.5700670

[24] Kluger MS, Shiao SL, Bothwell AL, Pober JS (2002) Cutting Edge: Internalization of transduced E-selectin by cultured human endothelial cells: comparison of dermal microvascular and umbilical vein cells and identification of a phosphoserine-type di-leucine motif. J Immunol 168: 2091–2095. 11859093 10.4049/jimmunol.168.5.2091

[25] Zheng L, Dengler TJ, Kluger MS, Madge LA, Schechner JS, et al (2000) Cytoprotection of human umbilical vein endothelial cells against apoptosis and CTL-mediated lysis provided by caspase-resistant Bcl-2 without alterations in growth or activation responses. J Immunol 164: 4665–4671. 10779771 10.4049/jimmunol.164.9.4665

[26] Heit B, Colarusso P, Kubes P (2005) Fundamentally different roles for LFA-1, Mac-1 and alpha4-integrin in neutrophil chemotaxis. J Cell Sci 118: 5205–5220. 16249234 10.1242/jcs.02632

[27] Huber AR, Kunkel SL, Todd RF, Weiss SJ (1991) Regulation of transendothelial neutrophil migration by endogenous interleukin-8. Science 254: 99–102. 1718038 10.1126/science.1718038

[28] Rot A, Jones AP, Webb LM (1993) Some aspects of NAP-1/IL-8 pathophysiology. II: Chemokine secretion by exocrine glands. Adv Exp Med Biol 351: 77–85. 7942300 10.1007/978-1-4615-2952-1_9

[29] Makó V, Czúcz J, Weiszhár Z, Herczenik E, Matkó J, et al (2010) Proinflammatory activation pattern of human umbilical vein endothelial cells induced by IL-1β, TNF-α, and LPS. Cytometry A 77: 962–970. 21290470 10.1002/cyto.a.20952

[30] Ding ZM, Babensee JE, Simon SI, Lu H, Perrard JL, et al (1999) Relative contribution of LFA-1 and Mac-1 to neutrophil adhesion and migration. J Immunol 163: 5029–5038. 10528208

[31] Nourshargh S, Marelli-Berg FM (2005) Transmigration through venular walls: a key regulator of leukocyte phenotype and function. Trends Immunol 26: 157–165. 15745858 10.1016/j.it.2005.01.006

[32] Woodfin A, Voisin MB, Imhof BA, Dejana E, Engelhardt B, et al (2009) Endothelial cell activation leads to neutrophil transmigration as supported by the sequential roles of ICAM-2, JAM-A, and PECAM-1. Blood 113: 6246–6257. 19211506 10.1182/blood-2008-11-188375 PMC2699241

[33] Burns AR, Smith CW, Walker DC (2003) Unique structural features that influence neutrophil emigration into the lung. Physiol Rev 83: 309–336. 12663861 10.1152/physrev.00023.2002

[34] Ley K, Laudanna C, Cybulsky MI, Nourshargh S (2007) Getting to the site of inflammation: the leukocyte adhesion cascade updated. Nat Rev Immunol 7: 678–689. 17717539 10.1038/nri2156

[35] Maier CL, Pober JS (2011) Human placental pericytes poorly stimulate and actively regulate allogeneic CD4 T cell responses. Arterioscler Thromb Vasc Biol 31: 183–189. 21051666 10.1161/ATVBAHA.110.217117 PMC3380367

[36] Bixel MG, Li H, Petri B, Khandoga AG, Khandoga A, et al (2010) CD99 and CD99L2 act at the same site as, but independently of, PECAM-1 during leukocyte diapedesis. Blood 116: 1172–1184. 20479283 10.1182/blood-2009-12-256388

[37] Bixel MG, Petri B, Khandoga AG, Khandoga A, Wolburg-Buchholz K, et al (2007) A CD99-related antigen on endothelial cells mediates neutrophil but not lymphocyte extravasation in vivo. Blood 109: 5327–5336. 17344467 10.1182/blood-2006-08-043109

[38] Huang AJ, Furie MB, Nicholson SC, Fischbarg J, Liebovitch LS, et al (1988) Effects of human neutrophil chemotaxis across human endothelial cell monolayers on the permeability of these monolayers to ions and macromolecules. J Cell Physiol 135: 355–366. 3397383 10.1002/jcp.1041350302

[39] Komatsu M, Tatum L, Altman NH, Carothers Carraway CA, Carraway KL (2000) Potentiation of metastasis by cell surface sialomucin complex (rat MUC4), a multifunctional anti-adhesive glycoprotein. Int J Cancer 87: 480–486. 10918186 10.1002/1097-0215(20000815)87:4<480::aid-ijc4>3.0.co;2-6

